# Thyroid Autoimmunity During Universal Salt Iodisation—Possible Short-Term Modulation with Longer-Term Stability

**DOI:** 10.3390/nu16244299

**Published:** 2024-12-12

**Authors:** Navoda Atapattu, Renuka Jayatissa, Harendra de Silva, Mohamed A. Adlan, Emmanuel K. Obuobie, Lakdasa D. Premawardhana

**Affiliations:** 1 Departments of Paediatrics and Paediatric Endocrinology, Lady Ridgeway Hospital, Colombo 08, Sri Lanka; navodaa@gmail.com (N.A.); harendra51@gmail.com (H.d.S.); 2 Faculty of Food and Nutrition, International Institute of Health Sciences, Colombo 12, Sri Lanka; renukajayatissa@ymail.com; 3 Department of Endocrinology and Diabetes, Aneurin Bevan University Health Board, Newport NP20 2UB, UK; mohamed.adlan@wales.nhs.uk (M.A.A.); kofi.obuobie@wales.nhs.uk (E.K.O.); 4 Thyroid Research Group, Division of Infection and Immunity, Cardiff University School of Medicine, Cardiff CF14 4XN, UK

**Keywords:** universal salt iodisation, thyroid autoimmunity, TPOAb, TgAb

## Abstract

Universal salt iodisation (USI) plays an essential role in the provision of iodine (I) to populations worldwide. Countries adopting USI programmes, adhering to strict criteria laid down by expert organisations such as the Iodine Global Network, are estimated to have reduced the prevalence of I deficiency by 75% (protecting 720 million individuals worldwide). Despite this success, doubts have been raised as to the desirability of continuing such programmes because of (a) the need to reduce salt intake for cardiovascular prevention and (b) the induction of thyroid autoimmunity. We present current evidence from cross-sectional studies in several disparate populations of the possible short-term modulation of thyroid autoimmune markers, thyroid peroxidase (TPOAb) and thyroglobulin antibodies (TgAb), with minimal disruption of biochemical thyroid function. We also present evidence from longer term, mainly cross-sectional studies, that indicate a reduction in the prevalence of TPOAb and TgAb, and the persistence of normal biochemical thyroid function over as long as two decades of USI. We believe these studies indicate that USI is safe, and that long-term salt iodisation does not cause an increase in autoimmune thyroid disease in the populations studied and should not be a safety concern based on current evidence. More long-term and better-designed studies are required.

## 1. Introduction

Iodine (I) is a micronutrient essential for human growth, development and organ function, all of which are mediated through its role in thyroid hormone synthesis [1,2,3,4]. The thyroid produces two iodinated hormones, and this synthetic function depends on a regular and adequate I supply. The current primary source of I in many regions of the world is iodised salt obtained through national universal salt iodisation (USI) schemes introduced over the last decades [4]. The WHO recommends a median urine I concentration between 100 and 199 μg/L as “adequate” I nutrition in community assessments of the population [5]. Recent surveys indicate a variable coverage of salt iodisation in large parts of the world, with deficient coverage in some, particularly in the East Asia region [6]. I obtained from dietary sources is reduced to iodide after ingestion and is absorbed through the stomach and duodenal mucosa utilising the sodium iodide symporter mechanism present on the basolateral plasma membrane of small-bowel enterocytes [3,7,8]. Absorbed I is largely stored in the thyroid and later used for the synthesis of triiodothyronine (T3) and thyroxine (T4) controlled by the hypothalamo–pituitary–thyroid control mechanism [3].

I deficiency may manifest itself clinically from foetal to adult life and cause stillbirth, irreversible brain damage, intellectual impairment, impaired growth, goitre etc. [9,10,11,12,13]. Although national USI schemes have ameliorated most of the above in countries where it has been successfully adopted, constant monitoring is essential to prevent both I undernutrition or excess. It has latterly been shown that the relationship between I nutrition and thyroid dysfunction is a U-shaped one: both the excess and deficiency of I may result in biochemical thyroid dysfunction, including thyroid autoimmunity, even when the influence of other factors have been eliminated [12]. In this regard, the early “peak” of thyroid autoimmune markers seem to subside with continuing and optimal I nutrition over many years [14,15]. The contribution of co-existent autoimmune disease, population genetics and other nutritional factors (e.g., selenium) need to be considered when attributing cause and effect to suboptimal I nutrition particularly in the development of thyroid autoimmunity [4,16]. Selenium deficiency has been highlighted as a contributor to thyroid autoimmune disease in this regard in large population studies and will need to be investigated more fully regarding its contribution to the manifestations of suboptimal I nutrition [17]. Thyroid autoimmunity may manifest itself as a high prevalence of thyroid autoimmune markers, e.g., TPOAb and TgAb, or as an increased prevalence of thyroid dysfunction in the form usually of overt or subclinical hypothyroidism [18]. While abnormal thyroid “function” (both hypothyroidism and hyperthyroidism) can result from suboptimal I nutrition, we shall concentrate on the available evidence and possible mechanisms for the potential modulation of thyroid autoimmunity with continuing I intake. We wish to reiterate that correctly administered USI remains a cornerstone of global public health, of which the benefits far outweigh its potential and hitherto unclear disadvantages [5].

## 2. Iodine and Thyroid Autoimmunity—Evidence

Studies reporting a relatively early change (within 5–8 years) to thyroid autoimmunity (and indeed subclinical hypothyroidism) after USI have been carried out mainly in Denmark [14,19,20,21], Sri Lanka [22,23,24] and China [25,26,27,28] (Table 1). These are mainly cross-sectional in design, with only one small cohort being followed up in Sri Lanka for a few years [23]. They showed an increased prevalence of thyroid antibodies and evidence of thyroid dysfunction in a minority. However, studies undertaken many years or a few decades after USI (see below) from Sri Lanka, Italy, Australia and China (all between 15 and 20 years after USI) have largely shown an approximate return to pre-USI levels of thyroid antibody prevalence and biochemical thyroid function; data are however, incomplete from some of these regions for comparison with previous years, and for providing incontrovertible evidence for modulation of autoimmune markers.

### 2.1. Evidence from Short-Term Studies

(i) Four-to-five years after the introduction of USI in Denmark, the prevalence of TPOAb increased from 14.3% to 23.8%, and the prevalence of TgAb increased from 13.7% to 19.9% [14]. The increase was greatest in young women and only high levels of TPOAb were linked to thyroid dysfunction, i.e., high TSH levels [14]. A similar increase in TPOAb and TgAb prevalence occurred in pregnant women over a period of 10 years, without a change in thyroid function [21] These findings are consistent with the DanThyr data [20,29].
(ii) Our own studies of schoolchildren in Sri Lanka have shown interesting results: (a) there was a high TgAb prevalence of 42.1% and a TPOAb prevalence of 8.7% about 5 years after USI was introduced, with a 1.1% prevalence of subclinical hypothyroidism in female schoolchildren [22]. This high prevalence of thyroid antibodies was predominantly in high-endemic-goitre areas denoting a predilection for areas with high levels of I deficiency. (b) Three years later, a further study in schoolgirls revealed a lower prevalence of thyroid antibodies despite unchanged (and satisfactory) urinary iodine concentrations. The prevalence of TgAb alone was 34%, TPOAb alone was 18.2%, and combined TPOAb and TgAb prevalence was 46.9% indicating a decrease in TgAb prevalence and a mild increase in TPOAb prevalence. The prevalence of subclinical hypothyroidism increased slightly too (2.1%). There was a lower mean thyroid volume and goitre prevalence in the second study (both when thyroid volume in relation to age and body surface area were concerned) [23]. (c) Of the forty-two schoolgirls followed up from the previous study (81% with TgAb, 19% with TPOAb initially), TgAb had become negative in 84% of the previously TgAb positive individuals, but 21.4% were TPOAb-positive. However, all these individuals had normal biochemical thyroid function [23].
(iii) Similar patterns have emerged from China [25,26,27,28]. In the largest such study conducted by Li et al. [27] a baseline prevalence of TPOAb and TgAb of 9.81 and 9.09%, respectively, (higher prevalence in women, older individuals and in areas with high I intake) was found. The follow-up cumulative incidence 5 years later was TPOAb 2.92% and TgAb 3.87%. The increase in S-Hypo was only seen in areas with more-than-adequate/excess iodine intake. The authors of these studies contend that an adequate intake of I is safe in terms of the development of thyroid autoimmunity, but both I deficiency and I excess are associated with increased thyroid autoantibody prevalence and AITD (Table 1).

### 2.2. Evidence from Long-Term Studies

However, studies carried out after over a decade or more of USI have indicated that population thyroid autoimmune parameters were stable after USI for many years, extending even up to 20 years.

(i) A large well-conducted retrospective study from Tasmania of more than 350,000 individuals showed that between 1995 and 2013, the (i) median urine iodine content increased from 75 to 108 μg/L; (ii) there was no significant increase in TPOAb over time (18.6% vs. 21.6% in those <40 years of age; 28.7% vs. 28.1% > 40 years pre- and post-USI, respectively); and (iii) there was a decreasing trend in overt thyroid dysfunction, after the introduction of USI [30].
(ii) A study from Northeast Germany (population recruited 7 and 17 years after USI) found that median urinary iodine concentrations decreased from 123 to 112 μg/L between 2000 and 2010, and thyroid autoimmune markers were stable with a decreased prevalence of goitre [31]. However, the prevalence of known thyroid disorders increased from 7.6 to 18.9%. The prevalence of positive TPOAb decreased from 3.9 to 2.9% (*p* = 0.022), and the prevalence of goitre decreased from 35.1 to 29.4% (*p* < 0.001).
(iii) In the Pescapagano survey in Italy (15 years after USI was introduced), there was an increase in thyroid autoimmunity in both males and females, with an increase in TgAb, which was more evident in those with ultrasound evidence of thyroiditis [32,33]. TgAb and TPOAb were present in 12.6% (age-related increase from 2.4% in children to 21.9% in the 46–55-year-old groups). More frequent in goitrous than non-goitrous individuals (14.6% vs. 5.9%).
(iv) Sixteen years after USI was introduced, a study from China [34] indicated that the prevalence of TPOAb was 9.8% and TgAb was 9.1 of %. This increased to a prevalence of TPOAb of 12.6% and TgAb of 11.5% in 2010. But in another study, 20 years after USI, the prevalence of TPOAb was 9.7% and TgAb 10.2% [27], indicating relative stability after many years of USI. China has eliminated I deficiency in 28 of its provinces and is on the way to eliminating I deficiency in a further 4 [15]. Furthermore, there is new evidence of the influence of selenium on the prevalence of thyroid autoimmunity in China, which needs to be confirmed in studies from other areas [35].
(v) In Sri Lanka, with continuing stable and adequate I nutrition 20 years after the introduction of USI (median urine iodine 138.5 μg/L, IQR 79.4, 219), TgAb prevalence was 6.4% with a persistently but minimally elevated TPOAb of 10.3% and a prevalence of subclinical hypothyroidism of 3% using population-derived reference ranges [36]. The strongest association with biochemical thyroid dysfunction was in those with TPOAb concentrations of more than four times the upper limit of the reference range (33.8% of those who had TPOAb). There had, therefore, been a modulation of thyroid autoantibody profiles to a more “benign” pattern of thyroid autoimmunity, which is reassuring [22,36]
(vi) A recent analysis of the NHANES data from the USA [37] indicated that high urine iodine concentrations (500–800 μg/L) were associated with a higher risk of TPOAb positivity (OR 1.57; 1.07–2.3), TgAb positivity (OR 2.00; 1.1–3.65), and a higher thyroid autoimmune disease risk (62%) compared to those with a urine iodine of 100–200 μg/L. However, the authors showed a trend towards a U-shaped relationship between urine iodine and the risk of developing TPOAb. They also showed that these risks were modulated by the selenium content in the blood with this association disappearing at high selenium levels [37].

### 2.3. Thyroid Ultrasound Echogenicity as a Marker of Autoimmune Destruction

Studies that have investigated thyroid ultrasound hypoechogenicity as a marker of autoimmune destruction yielded mixed results—(a) a study from Brazil, showed increased hypoechogenicity of the thyroid in children when urine iodine was more than 300 μg/L and a decrease in hypoechogenicity when mean urine iodine was 165 μg/L [38]; (b) a study from Italy confirmed moderate or marked hypoechogenicity of the thyroid in 6.6% children whose median urine iodine was 129 μg/L (iodine sufficient) compared to a prevalence of 10.9% in areas where median urine iodine was 89 μg/L [39]; (c) in Liguria (Italy) where comparison was made with data collected 8 years previously (2 years after USI was undertaken), there was no increase in thyroid hypoechogenicity—3.3% vs. 2.7% [40]. These studies confirm the lack of thyroid damage as evidenced by thyroid hypoechogenicity after USI when adequate iodoprophylaxis is achieved [40].

## 3. Iodine and Thyroid Autoimmunity

### 3.1. Thyroid Antigens and Antibodies

The predominant antigens involved in thyroid autoimmunity are thyroid peroxidase (TPO), thyroglobulin (Tg) and the TSH receptor (TSHR). The role of the sodium iodide symporter (NIS) and pendrin (Pen) in the thyroid autoimmune process has not hitherto been delineated accurately [41].

TPO is a membrane-bound intracellular antigen [42]. Polyclonal TPOAb, mainly of IgG1 and IgG4 varieties [43], are directed towards the same TPO epitopes in normal individuals and thyroid disease. Tg contains many epitopes, out of which only a few are immunogenic [44,45]. TgAb found in healthy individuals are polyclonal usually and those found in AITD are oligoclonal and directed against specific epitopes [46]. It is also known that even within the different AITDs, TgAb subclasses were variable: IgG4 predominant in Graves’ disease (GD) and IgG2 predominant in Hashimoto’s thyroiditis (HT) [46].

Although autoimmune thyroid antibodies usually correlate well with the degree of thyroid “damage” in AITD [47], they may also appear in serum with minimal histological damage to the thyroid gland [48]. The effect of salt iodisation and increasing population I levels on their prevalence and its modulation has been discussed above.

### 3.2. Thyroid Peroxidase Antibodies (TPOAb)

Thyroid peroxidase is an enzyme closely related to thyroid hormone synthesis [49]. It is also an autoantigen to which antibodies (TPOAb) are directed in autoimmune thyroid disease, AITD [50]. While almost all subjects with HT and about 75% of subjects with GD have TPOAb, it is also known that their presence in unaffected individuals may portend future thyroid disease [50]. The mechanisms by which TPOAb are involved in thyroid autoimmunity involve causing thyroid cell death via antibody-dependent cytotoxic cells (ADCC), and C3 complement mediated cell death [51]. TPOAb may also contribute to thyroid autoimmunity by influencing the T-cell epitope repertoire [52].

### 3.3. Thyroglobulin Antibodies (TgAb)

Thyroglobulin is an iodinated protein and is the most abundant antigen in the thyroid gland, forming the matrix for thyroid hormone synthesis and storage [53,54] TgAb are found both in HT and euthyroid normal subjects, and seem to be less predictive of AITD than TPOAb. However, the epitopic patterns of subjects with autoimmunity-induced HT are different to those in normal subjects (as discussed above) and may help in identifying “pathogenic” TgAb, signifying autoimmune thyroid perturbations in them [55].

### 3.4. Sodium Iodide Symporter (NISAb) and Pendrin Antibodies (PenAb)

The role of NISAb and PenAb is still to be accurately delineated. Although NISAb is found in GD specially, its role in thyroid autoimmunity is unclear [56,57].

### 3.5. Possible Mechanisms

The exact role of I in thyroid autoimmunity induction is still debated and remains unsettled [58]. The likely possibilities are as follows:

(i) A direct influence on immune effector cells initiating and propagating thyroid autoimmunity.: this is characterized by increased thyroid lymphocyte infiltration, MHC Class II expression on thyrocytes, increased cytokine secretion and increased thyroid antibody secretion [58]. Thyroid immunity can be ameliorated by depleting immune effector cells CD4^+^ and CD8^+^ T cells or B cells, adding further credence to this possibility [59,60]. Animal experiments have shown that I treatment increases intracellular adhesion molecule–1 (ICAM-1) concentrations within the thyroid [61], enhancing immunocompetent lymphocyte infiltration into the thyroid. This holds true for other cytokines too.
(ii) An indirect mechanism whereby the possible effects on thyroid tissue produce secondary immune effects: the role of a sudden change in I nutrition and the induction of free radicals in producing thyroid damage have been invoked as a possible mechanism for the above [57,62].
(iii) Increasing I has also been shown to increase the immunogenicity of thyroglobulin (Tg). Early in vitro and animal experiments have demonstrated this phenomenon [61,62]. It has been shown that increasing Tg iodination uncovers normally hidden or “cryptic” epitopes which are highly immunogenic and leads to increased TgAb formation. This phenomenon is likely when I nutritional status changes in humans upon the introduction of salt iodisation and was confirmed in early studies from Italy.

## 4. Conclusions

USI mandates that all salt used for household food and processed food preparation is iodised within strictly specified criteria [63,64]. This programme adopted by many governments throughout the world has ensured an adequate supply of I to growing infants, children, pregnant and lactating mothers; perhaps the most important group with regards to iodine supplementation with the aim of preserving adequate cerebral function and intelligence [65]. It is estimated that USI has prevented an estimated 720 million cases of iodine deficiency—a 75% reduction from predicted figures [65]—and has proven to be highly cost effective. This is a major public health achievement and should be encouraged to continue, particularly in the prevention of childhood iodine-deficiency-related disease. However, it is estimated that 1.88 billion people across the world remain at the risk of iodine deficiency [64].

**Figure 1:**
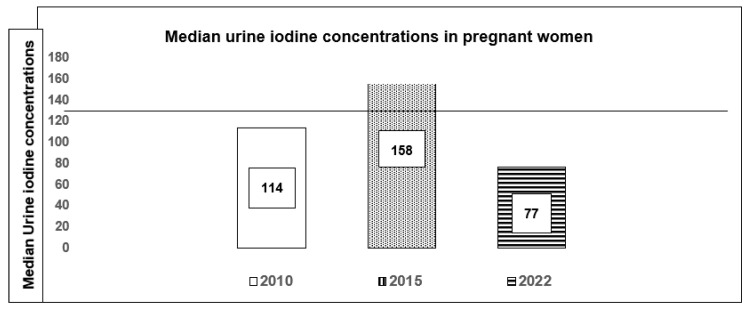
Median urine iodine concentrations in pregnant women in Sri Lanka in surveys carried out in 2010, 2015 and 2022 (adapted from reference [66]).

Despite the above facts, there are “threats” to the continued implementation of USI from several angles. Amongst them is (a) the recent drive to limit salt consumption (in turn to reduce hypertension, cardio- and cerebrovascular disease) [64] and (b) evidence suggesting possible initiation of thyroid autoimmunity. We contend that although there maybe evidence for an increased prevalence of thyroid autoimmune markers in the early years after USI, the studies that demonstrated this phenomenon were small cross-sectional studies, using variable methodology (e.g., in the demographic characteristics of the populations studied and the biochemical and immunological assays used), with a very limited follow up of affected cohorts. Furthermore, there is also good evidence that this initial “spike” seems to settle with a return to pre-USI levels, and long-term studies have confirmed this fact. Supporting USI programmes is essential to prevent a breakdown in the supply of iodine to vulnerable groups, as shown in studies in pregnant women from Sri Lanka: a median UIC which increased from 114 (59.5–193.1) μg/L in 2010 to 158 (90.6–256.3) μg/L in 2015, but declined to 77 (38.8–141.8) μg/L in 2022 (Figure 1) [66].

## Figures and Tables

**Table 1:** Studies related to early autoimmune thyroid dysfunction after USI.

Author, Year, (Ref.), n = Number of Participants	Country	Study Population	Post-USI (Years)	Changes in Pattern of Thyroid Disorders
Laurberg, 2006 [13] n = 4649	Denmark	Community-dwelling population sampled from two areas with different iodine intakes: Aalborg and Copenhagen	4–5	(1) UI 53 LL (Aalborg) and 68 μg/L (Copenhagen) in the two areas (2) TgAb prevalence at 13.0%; TPOAb prevalence at 13.1% (3) Increased incidence of hyperthyroidism in old and young (4) (Summary antibody titers or *p* values were not given in this study)
Pedersen, 2011 [14] n = 4649 + 3570	Denmark	Community-dwelling population sampled from two areas with different iodine intake before and after USI Females—3712 (19–65 years); Males—937 (61–65 years)	4–5	(1) TPOAb prevalence (defined as TPOAb > 30 U/mL,) was 14.3% before USI and increased to 23.8% after USI (*p*< 0.001) TgAb prevalence (defined as TgAb > 20 U/mL) was 13.9% and increased to 19.9% (*p* < 0.001) (2) Pronounced in young women (18–45 years) and mostly at low concentrations N.B. When the cutoff for antibody positivity was increased to >60 U/mL The prevalence of TPOAb was significantly lower: 11. 4 vs. 14.1%, *p* < 0.001; and there was no increase in TgAb prevalence: 7.9% vs. 8.6%, *p* = 0.158.
Premawardhana, 2000 [22] n = 367	Sri Lanka	Female schoolchildren aged 11–16 years from three areas with different endemic goitre prevalence	5	(1) TPOAb (>19.4 kIU/L) prevalence <10% in all ages (2) TgAb (>98 kIU/L) prevalence 14.3% in 11 years, 19.5% in 12 years, 44.1% in 13 years, 53.0%in 14 years, 52.0% in 15 years and 69.7% in 16 years old schoolchildren (3) TgAb detected at low concentrations and mostly in areas with the highest prevalence of goitre (4) No overt thyroid dysfunction
Mazziotti, 2003 [23] n = 282	Sri Lanka	a. Female schoolchildren aged 11–17.5 years from three areas with different endemic goitre prevalence	8	TgAb (>98 kIU/L) and TPOAb (>19.4 kIU/L)— (1) TgAb prevalence 34.8% (2) TgAb + TPOAb prevalence 46.9% (3) Reduced TgAb prevalence (from up to 70% to about 40%) (4) Increased TPOAb prevalence (from < 10% to 18.6%) (5) Increased subclinical hypothyroidism (defined as TSH > 5.2 mIU/L) from 1.0% to 6.3%) (6) No overt thyroid dysfunction—reference ranges for FT3 3.5–6.8 pmol/L; fT4 9.8–23 pmol/L and TSH 0.35–5.2 mU/L
		b. Follow up of 42 schoolchildren from previous study: 3 years later		(1) Reduced thyroid antibody prevalence—TgAb + TPOAb 23.8% vs. 46.9% 5 years before (2) Normal biochemical thyroid function
Li, 2008 [28] n = 3018 baseline study and 2381 follow up study in 5 years	China	Individuals aged more than 13 years from three communities with different levels of iodine intake Unselected adult subjects; Baseline study: females—2827; males—934 Follow up study: females—1748; males—633	3–8	(1) TPOAb and TgAb (more frequent in women and in areas with higher I intake) was 9.81 and 9.09%, respectively, at baseline (higher in older individuals) (reference ranges for TPOAb and TgAb are 35 and 40 IU/mL, respectively, and TSH (0.3–4.8 mIU/L) (2) Follow up cumulative incidence was TPOAb 2.9%; TgAb 3.9% (3) Increase of S-Hypo only in area with more than adequate/excess iodine intake (median UI 150 g/L) (4) The percentage of elevated TSH (TSH > 4.8 mIU/L) in either TPOAb or TgAb-positive subjects was 14.8%, and 2.7% in the antibody negative group (*p* < 0.0001) (5) The percentage of subnormal TSH (TSH < 0.3 mIU/L) in either TPOAb or TgAb-positive subjects was 15.3%, and 3.3% in the antibody-negative group (*p* < 0.0001)

The above studies show early thyroid autoimmune perturbations after the introduction of national schemes of USI. While changes occurred to the markers of thyroid autoimmunity (TgAb and TPOAb), overt or subclinical thyroid dysfunction was rare (see text). UI—urine iodine; USI—Universal Salt Iodisation; FT3—free triiodothyronine; FT4—free thyroxine; TSH—thyroid stimulating hormone.

## Data Availability

There were no new data created for this paper.

## References

[1] Klein K., Ojamaa J. (2001). Thyroid hormone and the cardiovascular system. N. Engl. J. Med..

[2] Jabbar A., Pingitore A., Pearce S.H.S., Zaman A., Iervasi G., Razvi S. (2017). Thyroid hormones and cardiovascular disease. Nat. Rev. Cardiol..

[3] Zimmermann M.B., Boelaert K. (2015). Iodine deficiency and thyroid disorders. Lancet Diabetes Endocrinol..

[4] Bath S.C. (2024). Thyroid function and iodine intake: Global recommendations and relevant dietary trends. Nat. Rev. Endocrinol..

[5] WHO (2007). Assessment of Iodine Deficiency Disorders and Monitoring Their Elimination.

[6] Zimmermann M.B. (2020). Iodine and the iodine deficiency disorders. Present Knowledge in Nutrition.

[7] Lisco G., De Tullio A., Triggiani D., Zupo R., Giagulli V.A., De Pergola G., Piazzolla G., Guastamacchia E., Sabbà C., Triggiani V. (2023). Iodine Deficiency and Iodine Prophylaxis: An Overview and Update. Nutrients.

[8] Ravera S., Reyna-Neyra A., Ferrandino G., Amzel L.M., Carrasco N. (2017). The Sodium/Iodide Symporter (NIS): Molecular Physiology and Preclinical and Clinical Applications. Annu. Rev. Physiol..

[9] Wu Z., Liu Y., Wang W. (2024). The burden of iodine deficiency. Arch. Med. Sci..

[10] Pearce E.N., Pino S., He X., Bazrafshan H.R., Lee S.L., Braverman L.E. (2004). Sources of dietary iodine: Bread, cows’ milk, and infant formula in the Boston area. J. Clin. Endocrinol. Metab..

[11] Dunn J.T., Delange F. (2001). Damaged reproduction: The most important consequence of iodine deficiency. J. Clin. Endocrinol. Metab..

[12] Wang B., He W., Li Q., Jia X., Yao Q., Song R., Qin Q., Zhang J.-A. (2019). U-shaped relationship between iodine status and thyroid autoimmunity risk in adults. Eur. J. Endocrinol..

[13] Laurberg P., Jørgensen T., Perrild H., Ovesen L., Knudsen N., Pedersen I.B., Rasmussen L.B., Carle A., Vejbjerg P. (2006). The Danish investigation on iodine intake and thyroid disease, DanThyr: Status and perspectives. Eur. J. Endocrinol..

[14] Pedersen I.B., Knudsen N., Carlé A., Vejbjerg P., Jørgensen T., Perrild H., Ovesen L., Rasmussen L.B., Laurberg P. (2011). A cautious iodization program bringing iodine intake to a low recommended level is associated with an increase in the prevalence of thyroid autoantibodies in the population. Clin. Endocrinol..

[15] Sun D., Codling K., Chang S., Zhang S., Shen H., Su X., Chen Z., Scherpbier R.W., Yan J. (2017). Eliminating Iodine Deficiency in China: Achievements, Challenges and Global Implications. Nutrients.

[16] Pedersen I.B., Laurberg P., Knudsen N., Jørgensen T., Perrild H., Ovesen L., Rasmussen L.B. (2007). An increased incidence of overt hypothyroidism after iodine fortification of salt in Denmark: A prospective population study. J. Clin. Endocrinol. Metab..

[17] Wang F., Li C., Li S., Cui L., Zhao J., Liao L. (2023). Selenium and thyroid disease. Front. Endocrinol..

[18] Liu J., Feng Z., Gao R., Liu P., Meng F., Fan L., Liu L., Du Y. (2024). Analysis of risk factors for autoimmune thyroid disease based on blood indicators and urinary iodine concentrations. Front. Endocrinol..

[19] Vejbjerg P., Knudsen N., Perrild H., Carle A., Laurberg P., Pedersen I.B., Rasmussen L.B., Ovesen L., Jorgensen T. (2007). Effect of a mandatory iodization program on thyroid gland volume based on individuals’ age, gender, and preceding severity of dietary iodine deficiency: A prospective, population-based study. J. Clin. Endocrinol. Metab..

[20] Petersen M., Pedersen I.B., Knudsen M., Andersen S., Jørgensen T., Perrild H., Ovesen L., Rasmussen L.B., Thuesen B.H., Carlé A. (2019). Changes in subtypes of overt thyrotoxicosis and hypothyroidism following iodine fortification. Clin. Endocrinol..

[21] Bliddal S., Borresen S.W., Feldt-Rasmussen U. (2017). Increase in thyroglobulin antibody and thyroid peroxidase antibody levels, but not preterm birth-rate, in pregnant Danish women upon iodine fortification. Eur. J. Endocrinol..

[22] Premawardhana L.D., Parkes A.B., Smyth P.P., Wijeyaratne C.N., Jayasinghe A., de Silva D.G., Lazarus J.H. (2000). Increased prevalence of thyroglobulin antibodies in Sri Lankan schoolgirls-is iodine the cause?. Eur. J. Endocrinol..

[23] Mazziotti G., Premawardhana L.D., Parkes A.B., Adams H., Smyth P.P., Smith D.F., Kaluarachi W.N., Wijeyaratne C.N., Jayasinghe A., de Silva D.G. (2003). Evolution of thyroid autoimmunity during iodine prophylaxis. The Sri Lankan experience. Eur. J. Endocrinol..

[24] Premawardhana L.D.K.E., Parker A.R., Mazziotti G., Lazarus J.H. (2003). Autoimmune thyroiditis after elimination of iodine deficiency in Sri Lanka. Thyroid.

[25] Teng W., Shan Z., Teng X., Guan H., Li Y., Teng D., Jin Y., Yu X., Fan C., Chong W. (2006). Effect of iodine intake on thyroid diseases in China. N. Engl. J. Med..

[26] Shan Z., Chen L., Lian X., Liu C., Shi B., Shi L., Tong N., Wang S., Weng J., Zhao J. (2016). Iodine status and prevalence of thyroid disorders after introduction of mandatory universal salt iodization for 16 years in China: A cross-sectional study in 10 Cities. Thyroid.

[27] Li Y., Teng D., Ba J., Chen B., Du J., He L., Lai X., Teng X., Shi X., Li Y. (2020). Efficacy and safety of long-term universal salt iodization on thyroid disorders: Epidemiological evidence from 31 provinces of Mainland China. Thyroid.

[28] Li Y., Teng D., Shan Z., Teng X., Guan H., Yu X., Fan C., Chong W., Yang F., Dai H. (2008). Antithyroperoxidase and antithyroglobulin antibodies in a five- year follow-up survey of populations with different iodine intakes. J. Clin. Endocrinol. Metab..

[29] Cerqueira C., Knudsen N., Ovesen L., Perrild H., Rasmussen L.B., Laurberg P., Jørgensen T. (2009). Association of iodine fortification with incident use of anti-thyroid medication—A Danish nationwide study. J. Clin. Endocrinol. Metab..

[30] Hong A., Stokes B., Otahal P., Owens D., Burgess J.R. (2017). Temporal trends in thyroid-stimulating hormone (TSH) and thyroid peroxidase antibody (ATPO) testing across two phases of iodine fortification in Tasmania (1995–2013). Clin. Endocrinol..

[31] Khattak R.M., Ittermann T., Nauck M., Below H., Völzke H. (2016). Monitoring the prevalence of thyroid disorders in the adult population of Northeast Germany. Popul. Health Metr..

[32] Aghini Lombardi F., Fiore E., Tonacchera M., Antonangeli M., Rago T., Frigeri M., Provenzale A.M., Montanelli L., Grasso L., Pinchera A. (2010). The effect of voluntary iodine prophylaxis in a small rural community: The Pescopagano survey 15 years later. J. Clin. Endocrinol. Metab..

[33] Latrofa F., Fiore E., Rago T., Antonangeli L., Montanelli L., Ricci D., Provenzale M.A., Scutari M.A., Frigeri M., Tonacchera M. (2013). Iodine contributes to thyroid autoimmunity in humans by unmasking a cryptic epitope on thyroglobulin. J. Clin. Endocrinol. Metab..

[34] Wan S., Jin B., Ren B., Qu M., Wu H., Liu L., Boah M., Shen H. (2020). The relationship between high iodine consumption and levels of autoimmune thyroiditis-related biomarkers in a Chinese population: A meta-analysis. Biol. Trace Elem. Res..

[35] Zhang C., Wang H., Teng W., Shan Z. (2024). The Relationships among the Urinary Iodine Concentration, Selenium Intake, and Thyroid Antibodies in Adults, Including the Interaction between Iodine and Selenium: National Health and Nutrition Examination Survey 2007–2012. Nutrients.

[36] Jayatissa R., Okosieme O.E., Ranasinghe S., Carter J.L., Gunatunga I.P., Lazarus J.H., Premawardhana L.D. (2021). Thyroid Autoimmunity and Dysfunction in Sri Lankan Children and Adolescents After 22 Years of Sustained Universal Salt Iodization. Thyroid.

[37] Miranda D.M., Massom J.N., Catarino R.M., Santos R.T., Toyoda S.S., Marone M.M., Tomimori E.K., Monte O. (2015). Impact of nutritional iodine optimization on rates of thyroid hypoechogenicity and autoimmune thyroiditis: A cross-sectional, comparative study. Thyroid.

[38] De Angelis S., Bagnasco M., Moleti M., Regalbuto C., Tonacchera M., Vermiglio F., Medda E., Rotondi D., Di Cosmo C., Dimida A. (2021). Obesity and Monitoring iodine nutritional status in schoolchildren: Is body mass index a factor to consider?. Thyroid.

[39] Teti C., Panciroli M., Nazzari E., Pesce G., Mariotti S., Olivieri A., Bagnasco M. (2021). Iodoprophylaxis and thyroid autoimmunity: An update. Immunol. Res..

[40] Bogusławska J., Godlewska M., Gajda E., Piekiełko-Witkowska A. (2024). Cellular and molecular basis of thyroid autoimmunity. Eur. Thyroid J..

[41] McLachlan S.M., Rapoport B. (1992). The molecular biology of thyroid peroxidase: Cloning, expression and role as autoantigen in autoimmune thyroid disease. Endocr. Rev..

[42] Xie L.D., Gao Y., Li M.R., Lu G.Z., Guo X.H. (2008). Distribution of immunoglobulin G subclasses of anti-thyroid peroxidase antibody in sera from patients with Hashimoto’s thyroiditis with different thyroid functional status. Clin. Exp. Immunol..

[43] Latrofa F., Ricci D., Grasso L., Vitti P., Masserini L., Basolo F., Ugolini C., Mascia G., Lucacchini A., Pinchera A. (2008). Characterization of thyroglobulin epitopes in patients with autoimmune and non-autoimmune thyroid diseases using recombinant human monoclonal thyroglobulin autoantibodies. J. Clin. Endocrinol. Metab..

[44] Volpe R., Volpe R. (1990). Immunology of human thyroid disease. Autoimmune Diseases of the Endocrine System.

[45] Caturegli P., Kuppers R.C., Mariotti S., Burek C.L., Pinchera A., Ladenson P.W., Rose N.R. (1994). IgG subclass distribution of thyroglobulin antibodies in patients with thyroid disease. Clin. Exp. Immunol..

[46] Arai T., Kurashima C., Utsuyama M., Sawabe M., Ito H. (2000). Measure- ment of anti-thyroglobulin and anti-thyroid peroxidase antibod- ies using highly sensitive radioimmunoassay: An effective method for detecting asymptomatic focal lymphocytic thyroiditis in the elderly. Endocr. J..

[47] Benvenga S., Trimarchi F. (2008). Changed presentation of Hashimoto’s thyroiditis in North-Eastern Sicily and Calabria (Southern Italy) based on a 31-year experience. Thyroid.

[48] Godlewska M., Banga P.J. (2019). Thyroid peroxidase as a dual active site enzyme: Focus on biosynthesis, hormonogenesis and thyroid disorders of autoimmunity and cancer. Biochimie.

[49] Godlewska M., Gawel D., Buckle A.M., Banga J.P. (2019). Thyroid peroxidase revisited—what’s new?. Horm. Metab. Res..

[50] Czarnocka B., Eschler D.C., Godlewska M., Tomer Y., Shoenfeld Y., Meroni P.L., Gershwin M.E. (2014). Chapter 44—Thyroid autoantibodies: Thyroid peroxidase and thyroglobulin antibodies. Autoantibodies.

[51] Godlewska M., Czarnocka B., Gora M. (2012). Localization of key aminoacid residues in the dominant conformational epitopes on thyroid peroxidase recognized by mouse monoclonal antibodies. Autoimmunity.

[52] Dubska M., Banga J.P., Plochocka D., Hoser G., Kemp E.H., Sutton B.J., Gardas A., Gora M. (2006). Structural insights into autoreactive determinants in thyroid peroxidase composed of discontinuous and multiple key contact amino acid residues contributing to epitopes recognized by patients’ autoantibodies. Endocrinology.

[53] Di Jeso B., Arvan P. (2016). Thyroglobulin from molecular and cellular biology to clinical endocrinology. Endocr. Rev..

[54] Mclachlan S.M., Rapoport B. (2017). Thyroid autoantibodies display both ‘original antigenic sin’ and epitope spreading. Front. Immunol..

[55] Frohlich E., Wahl R. (2017). Thyroid autoimmunity: Role of anti-thyroid antibodies in thyroid and extra-thyroidal diseases. Front. Immunol..

[56] Eleftheriadou A.M., Mehl S., Renko K., Kasim R.H., Schaefer J.A., Minich W.B., Schomburg L. (2020). Re-visiting autoimmunity to sodium-iodide symporter and pendrin in thyroid disease. Eur. J. Endocrinol..

[57] Luo Y., Kawashima A., Ishido Y., Yoshihara A., Oda K., Hiroi N., Ito T., Ishii N., Suzuki K. (2014). Iodine Excess as an Environmental Risk Factor for Autoimmune Thyroid Disease. Int. J. Mol. Sci..

[58] Hutchings P.R., Cooke A., Dawe K., Champion B.R., Geysen M., Valerio R., Roitt I.M. (1992). A thyroxine-containing peptide can induce murine experimental autoimmune thyroiditis. J. Exp. Med..

[59] Yu S., Dunn R., Kehry M.R., Braley-Mullen H. (2008). B cell depletion inhibits spontaneous autoimmune thyroiditis in NOD. H-2h4 mice. J. Immunol..

[60] Rasooly L., Rose N.R., Saboori A.M., Ladenson P.W., Burek C.L. (1998). Iodine is essential for human T cell recognition of human thyroglobulin. Autoimmunity.

[61] Carayanniotis G. (2003). The cryptic self in thyroid autoimmunity: The paradigm of thyroglobulin. Autoimmunity.

[62] Dai Y.D., Rao V.P., Carayanniotis G. (2002). Enhanced iodination of thyroglobulin facilitates processing and presentation of a cryptic pathogenic peptide. J. Immunol..

[63] Rigutto Fairbrother J., Zimmermann M.B. (2024). Salt Reduction and Iodine Fortification Policies Are Compatible: Perspectives for Public Health Advocacy. Nutrients.

[64] World Health Organization (2014). Guideline: Fortification of Food-Grade Salt with Iodine for the Prevention and Control of Iodine Deficiency Disorders.

[65] Gorstein J.L., Bagriansky J., Pearce E.N., Kupka R., Zimmermann M.B. (2020). Estimating the Health and Economic Benefits of Universal Salt Iodization Programs to Correct Iodine Deficiency Disorders. Thyroid.

[66] Jayatissa R., Perera A., Alwis N. (2023). National Nutrition and Micronutrient Survey in Sri Lanka: 2022.

